# The effects of 100% wild blueberry (*Vaccinium angustifolium*) juice consumption on cardiometablic biomarkers: a randomized, placebo-controlled, crossover trial in adults with increased risk for type 2 diabetes

**DOI:** 10.1186/s40795-017-0164-0

**Published:** 2017-05-25

**Authors:** K. S. Stote, M. I. Sweeney, T. Kean, D. J. Baer, J. A. Novotny, N. L. Shakerley, A. Chandrasekaran, P. M. Carrico, J. A. Melendez, K. T. Gottschall-Pass

**Affiliations:** 1 Division of Science, Mathematics and Technology, State University of New York, Empire State College, 113 West Avenue, Saratoga Springs, NY 12866 USA; 2 Departments of Biology (MIS), Applied Human Sciences (KTG) and School of Nursing (TK) University of Prince Edward Island, Charlottetown, Prince Edward Island Canada; 3 Beltsville Human Nutrition Research Center, US Department of Agriculture, Agriculture Research Service, Beltsville, USA; 4 Nanobioscience Constellation, State University of New York Polytechnic Institute, Albany, NY USA; 5 Department of Biology, State University of New York, University at Albany, Albany, USA

**Keywords:** 100% wild blueberry juice, Nitric oxide, Blood pressure, Endothelial function, Risk factors for type 2 diabetes

## Abstract

**Background:**

Wild blueberries have a high content of polyphenols, but there is limited data evaluating their health benefits in adults at risk for type 2 diabetes. The objective of the study was to investigate whether consumption of 100% wild blueberry juice improves cardiometabolic biomarkers associated with type 2 diabetes risk.

**Methods:**

A single-blind, randomized, placebo-controlled, crossover design trial was conducted in which adults (women, *n* = 19, ages 39–64 y) at risk for type 2 diabetes consumed 240 mL of wild blueberry juice or a placebo beverage as part of their free-living diet for 7 days. Blood was collected to determine various biomarkers such as fasting plasma glucose, fasting serum insulin, surrogate markers of insulin sensitivity, triglycerides, inflammation (interleukin-6, interleukin-10, high-sensitivity C-reactive protein, tumor necrosis factor-alpha, serum amyloid A), adhesion molecules (soluble intercellular adhesion molecule-1, soluble vascular adhesion molecule-1), oxidative stress (LDL-oxidation, total 8-isoprostanes), and nitric oxide. Endothelial function and blood pressure were also assessed.

**Results:**

Wild blueberry juice consumption for 7 days produced no significant changes in glucose, insulin, insulin sensitivity, triglycerides, inflammatory markers, adhesion molecules, oxidative stress, endothelial function or blood pressure. However, wild blueberry juice consumption showed a trend for lowering systolic blood pressure: 120.8 ± 2.2 mmHg in the placebo group vs 116.0 ± 2.2 mmHg in the blueberry juice group (*P* = 0.088). Serum concentrations of nitrates and nitrites, an index of nitric oxide production, increased from 2.9 ± 0.4 μM after placebo drink to 4.1 ± 0.4 μM after drinking wild blueberry juice (*P* = 0.039).

**Conclusions:**

Short-term consumption of wild blueberry juice may promote cardioprotective effects, by improving systolic blood pressure, possibly through nitric oxide production, in adults at risk for type 2 diabetes. This outcome warrants longer-term human studies of blueberries, including defined amounts of either the whole fruit or juice, to clarify whether polyphenol-rich foods can be efficacious for improving cardiometabolic biomarkers in adults at risk for type 2 diabetes.

**Trial registration:**

NCT02139878, clinicaltrials.gov; date of registration: May 4, 2014.

## Background

Diabetes mellitus and its sequelae are a major and growing public health problem. The prevalence of diabetes worldwide is greater than 371 million persons or approximately 7.5% of the population, and is projected to increase to 552 million by 2030. The number of persons with impaired glucose tolerance is estimated to increase as well. The development of type 2 diabetes is related to obesity, aging, and a sedentary lifestyle, and is a major risk factor for heart attack and stroke [1, 2].

Lifestyle strategies that include dietary modification, such as consuming a plant-based diet, are well recognized in disease prevention generally, and may improve risk factors for type 2 diabetes [3, 4]. Various bioactive components of a plant-based diet contribute to its beneficial health effects, but there has been keen interest in the possibility that plant polyphenols play a role. Blueberries are a rich dietary source of polyphenols, specifically anthocyanins, which make up >50% of total phenolic compounds in blueberries, followed by hydroxycinnamic acid derivatives, flavonols, and flavanols [5].

To date, there are few human clinical trials evaluating the beneficial health effects of blueberries in populations at risk for type 2 diabetes. Four randomized, parallel design human trials have reported that consumption of blueberries may beneficially affect insulin sensitivity and other early biomarkers of cardiovascular disease, such as blood pressure, endothelial function, and oxidative stress [6–9]. A systematic review has further concluded that there is a lack of clinical trials evaluating blueberry consumption in humans at risk for disease [10]. The prevalence of individuals at risk for type 2 diabetes is increasing in the population; thus, it is important to determine whether anthocyanin-rich foods can positively alter early biomarkers of cardiovascular disease and type 2 diabetes. Several prospective studies showed an inverse association between a higher intake of anthocyanins and risk of chronic diseases, such as type 2 diabetes [11, 12]. In addition, a recent evidence-based review concluded that there is a lack of well-designed clinical trials evaluating standardized doses or reasonable amounts of 100% fruit juice consumption in humans [13].

We conducted a randomized, single-blind human intervention trial with 100% wild blueberry juice, implementing a crossover design with a placebo control. We hypothesized that the intake of wild blueberry juice may have promising effects on cardiometabolic biomarkers in adults having ≥2 risk factors for type 2 diabetes. We also considered that short-term intake of wild blueberry consumption may produce possible differences in the study outcomes, due to results of previous acute human berry clinical trials [14–17]. Cardiometabolic biomarkers included blood pressure, endothelial function, insulin sensitivity, fasting plasma glucose, fasting serum insulin, triglycerides, inflammation (IL-6, IL-10, high-sensitivity CRP, TNF-α, SAA), adhesion molecules (sICAM, sVCAM), oxidative stress (LDL-oxidation, total 8-isoprostanes), and nitric oxide.

## Methods

### Subjects

Men and women aged 22 to 65 years were recruited by advertisement from the greater Charlottetown, Prince Edward Island, Canada area from March 2014 to May 2014. Inclusion in the study was based on having at least two risk factors for type 2 diabetes based on the Canadian Diabetes Association [18]. These risk factors included body mass index (BMI) >25 kg/m^2^; female waist circumference > 88 cm, male waist circumference > 102 cm; family history of type 1 or type 2 diabetes; history of gestational diabetes; a member of a type 2 diabetes high-risk population (Aboriginal, Hispanic, Asian, South Asian, or Africa decent); hypertension; and hyperlipidemia. Exclusion criteria included participants who: have a BMI <25 kg/m^2^; reported tobacco use; have given birth during the previous 12 months, are pregnant or planning to become pregnant during the study, lactating, or initiating or changing a hormone replacement therapy regimen within 3 months of the start of the study; a history or presence of kidney disease, liver disease, gout, certain cancers, thyroid disease, gastrointestinal disease, other metabolic diseases, or malabsorption syndromes; have type 1 and 2 diabetes requiring the use of oral anti-diabetic agents or insulin; have a history of eating disorders; have a history of drug and alcohol abuse; are routinely participating in a heavy exercise program or initiating an exercise program during the study; losing 10% of body weight within the past 12 months or planning to initiate weight loss; have a known (self-reported) allergy or adverse reaction to blueberry products. A nurse practitioner approved study entry based on the participants’ self-reported medical history, and review of current blood pressure and weight status. Participants gave their informed consent to participate, and the University of Prince Edward Island, Research Ethics Board approved the experimental protocol. The participants received $200 for their successful participation in the study. The trial was registered at clinicaltrials.gov (NCT02139878).

### Study design

The study was a randomized, single-blind, placebo-controlled, crossover design with 2 one-week treatment periods separated by an 8-day washout period. The randomization plan was generated with the use of the Second Generator Plan from randomization.com prior to the start of the study [19]. Participants were assigned to the randomization plan in order of recruitment. The principal investigator generated the randomization plan, and enrolled and assigned participants to the interventions. The study was conducted at the University of Prince Edward Island, Human Nutrition Research Center, Charlottetown, Prince Edward Island, Canada. Participants were randomly assigned to consume 240 mL wild blueberry juice or 240 mL placebo control beverage (color/flavor/energy-matched) per day along with their free-living diet. Investigators chose this amount of the 100% fruit juice because it is considered a reasonable amount of juice for participants to consume based on dietary guidance [13, 20, 21]. Participants who received the wild blueberry juice for the first week were given the placebo for the second week and vice versa. Beverage products were differentiated by labels marked in the colors green or yellow, but were otherwise identical in all other aspects including bottle shape, size, and color. Participants were not told which product was wild blueberry juice versus placebo until the end of the study. Participants were instructed to consume 120 mL of the beverage product with breakfast and the evening meal, for a total of 240 mL per day. A week’s supply of beverage products was provided in a cooler for the participants to pick up at the Human Nutrition Research Center each weekly visit. Participants were instructed to keep the beverage products refrigerated. In addition, participants were asked to rinse out each bottle with water and drink it to warrant full consumption of the beverage products. Compliance was determined by participants’ completion of a questionnaire each day, with a record of the time they consumed the beverage product; by participants’ verbal report of beverage consumption to study investigators; and by study investigators’ count of empty bottles returned weekly to the University of Prince Edward Island Human Nutrition Research Center.

### Wild blueberry juice and placebo

The blueberry juice used in this study was made using the wild (lowbush) blueberry variety, *Vaccinium angustifolium*. The wild blueberries were harvested in Tignish, Prince Edward Island, Canada, and processed into juice by the Prince Edward Island Juice Works, Prince Edward Island, Canada, in August 2013. The wild blueberry juice was stored at 4 °C until use. Samples of the juice were collected at the beginning of the study and at the end of the study in aliquots of 500 ml and stored at -20 °C until nutrient and polyphenol analysis. Proximate and sugar profile were determined by the Research and Productivity Council Science & Engineering, Fredericton, New Brunswick, Canada. Anthocyanins and total polyphenols were determined by the spectrophotometric pH differential and Folin–Ciocalteu method, respectively, at the University of Prince Edward Island Human Nutrition Research Center [22]. The placebo beverage was developed at the University of Prince Edward Island Human Nutrition Research Center to match sensory features similar to the wild blueberry juice but without polyphenols. The placebo beverage contained water, colorants (red and blue), artificial blueberry flavor, sucrose, fructose, alum, tartaric acid, and citric acid. Nutrient composition of the wild blueberry juice did not differ from the beginning of the study compared to the end of the study. Nutrient composition of the treatment beverages is shown in Table 1.

**Table 1:** Nutrient composition of wild blueberry juice and placebo treatment beverages^a^

	Treatment beverage	
	Wild blueberry Juice	Placebo
Energy, kcal	120	110
Fat, g	0	0
Protein, g	0	0
Sugars, g	30	27.5
Glucose, g	14	13
Fructose, g	16	14.5
Fiber, g	0	0
Total phenolics GAE, mg	2138	0
Total anthocyanins, mg	314	0

^a^Values represent content per 240 mL

### Dietary intake assessment

Participants were asked to eliminate consumption of anthocyanin-containing foods 7 days prior to starting the study and throughout the study duration. To achieve this, participants were asked to refrain from consuming berries and grapes or juices that contained them, as well as wine. Dietary intake data were collected and analyzed by ten 24-h dietary recalls throughout the study duration, and during the washout period, using the Automated Self-Administered 24-h Recall (ASA-24)-Canada-2014 system, developed by the Food Directorate at Health Canada in collaboration with the National Cancer Institute, Bethesda, MD. ASA24-Canada-2014 makes use of the ASA24, United States version, with adaptations made to reflect the Canadian food supply. Foods exclusive to Canada have been added, and those not available in Canada were removed. The ASA24 is an automated, multiple-pass, self-administered, online 24-h dietary recall method [23]. ASA24 has been validated in a small controlled feeding study and compared to intakes collected using the interviewer-administered USDA Automated Multiple-Pass Method [24–26]. At the first visit, participants received instruction about completing recalls from a registered dietitian and performed a practice recall. Every week, participants received an email and written instructions directing them to log on to a secure website and complete an ASA24-Canada-2014 recall for food consumed from midnight to midnight of the preceding day. The days of data collection ensured proportional representation of week and weekend days.

Participants completed a daily questionnaire regarding their general health; any consumption of prescription and over the counter medications; factors related to beverage and dietary compliance; and exercise performed. The questionnaire also gave participants the opportunity to write any questions regarding the study intervention. Participants were encouraged to maintain their normal exercise routine throughout the study. Few participants were on antihypertensive (*n* = 3) and lipid-lowering medication (*n* = 1); these participants were asked to maintain their same medication usage, dose, and timing without any change throughout the study. No participants were on hormone replacement therapy or oral contraceptives. In addition, participants were asked to abstain from using any non-prescription drugs, vitamin, and dietary supplements for at least 7 days prior to the start of the study and throughout the study duration.

### Body weight, body composition, and anthropometrics

Prior to the start and at the end of each treatment period, body weight and composition were measured using bioelectrical impedance analysis (model BF-350, Tanita, Arlington Heights, IL, USA). Measurements were made according to the manufacturer’s guidelines. Participants fasted for at least 12 h before the measurements were made and refrained from exercise. In addition, prior to the start of the intervention and at the end of each treatment period, waist circumference was measured above the right ilium and on the midaxillary line. Two trained individuals, according to a written protocol, took measurements with a fiberglass tape.

### Blood pressure and endothelial function

Resting blood pressure was measured in the morning, at the beginning and end of each treatment period according to a written protocol. Participants were instructed to refrain from consuming caffeine and were advised to empty their bladder prior to the measurement. Participants were asked to rest in a quiet, dimly lit room for 10 min. The appropriate size cuff was attached to the upper right arm according to the manufacturer’s instructions. An automatic cuff measured blood pressure 2 times with a 5-min interval (Omron, 7 Series, Omron Healthcare, Inc.). The mean of the 2 blood pressure measurements was used for statistical analysis.

Endothelial function was determined in the morning after a 12-h fast, at the beginning and the end of each treatment period (in a sitting position in a quiet, dimly lit, room) according to a written protocol. Measurements were done using the ENDO-PAT2000 device (Itamar Medical Ltd., Caesarea, Israel), a non-invasive test that records the peripheral artery tone (PAT) signal from biosensors placed on the subject’s index fingers. The signal was documented before and after inflating an occlusion blood pressure cuff on 1 arm. This process temporarily halts the blood flow to the index fingers. Computer software provided by the manufacturer calculates the reactive hyperemia index (RHI) which compares the arterial pressure ratio in the index fingers before and after occlusion. RHI is the post-to-pre occlusion PAT signal ratio in the occluded side, normalized to the control side and further corrected for baseline vascular tone; normal RHI is >1.67 while abnormal RHI is ≤1.67. RHI correlates with the gold standard method for the assessment of endothelial function, acetyl-choline infusion in coronary arteries, and also the ischemia-induced flow mediated dilation in the larger brachial artery measured by high-resolution ultrasound [27, 28].

### Biological sample collection and analysis

Blood was collected in the morning after a 12-h fast, at the beginning and end of each treatment period, by a registered nurse using sterile technique. The collected blood samples were used to prepare 0.8–2.0 mL aliquots of plasma (NaFl tubes) and serum that were stored at −80 °C until analysis. Serum concentrations of total, HDL and LDL cholesterol, triglyceride, and spectrophotometric procedures determined plasma glucose concentrations using an Abbott Aeroset Chemistry Analyzer (Queen Elizabeth Hospital, Charlottetown, Prince Edward Island, Canada). Serum insulin concentrations were measured by immunoassay with chemiluminescent detection on a Siemans Immulite 2000 XPi Immunoassay System and hemoglobin A1C was measured by using immunoturbidimetry (Queen Elizabeth Hospital, Charlottetown, Prince Edward Island, Canada). Plasma glucose, serum insulin, and triglyceride concentrations were used for the homeostasis model assessment of insulin resistance (HOMA-IR) and the quantitative insulin sensitivity check index (QUICKI). The equation used for QUICKI is 1/{[log(fasting insulin μU/ml) + log(fasting glucose mg/dL)]} and the equation used for HOMA-IR is 1/[glucose (mmol/L) X insulin (μU/L)/22.5] [29, 30]. Serum interleukin-6 (IL-6), interleukin-10 (IL-10), high sensitivity C-reactive protein (CRP), tumor necrosis factor-alpha (TNF-α), serum amyloid A (SAA), soluble intercellular adhesion molecule-1 (sICAM), and soluble vascular adhesion molecule-1 (sVCAM) were analyzed with a sandwich-type immunoassay method using eletrochemiluminescence detection (Meso Scientific Discovery) with analyses performed at the State University of New York, Polytechnic Institute, Albany, NY, USA. Analysis of LDL-oxidation (Mercodia 10–1143-01) was performed at the Food Components and Health Laboratory, Beltsville Human Nutrition Research, US Department of Agriculture, Beltsville, MD, USA. Total (free and esterfied) 8-isoprostane were measured at Cayman Chemical (Ann Arbor, MI, USA) using enzyme immunoassay. The ratio of nitrate to nitrite, an index of total nitric oxide, was determined at Cayman Chemical (Ann Arbor, MI, USA) using their nitrate/nitrite fluorometric assay. All analytes were measured in duplicate. The mean of the analytes was used for statistical analysis.

### Statistical analysis

Data were analyzed by analysis of covariance (ANCOVA) appropriate for a two-period, two-treatment crossover study with baseline values prior to each period (SAS, SAS Institute, Cary, NC, USA, version 9.4). The full statistical model included treatment sequence, period (first or second period), treatment group, and covariates BMI, weight at enrollment, height, age, percent body fat, waist circumference, insulin resistance status (HOMA-IR >2.6), blood pressure status (pre and stage 1 hypertension) and, where appropriate, systolic and diastolic blood pressure as fixed effects. Subject nested in sequence was included in the model as a random effect. Where the covariate was not statistically significant, it was dropped from the model. The relationship between outcome and treatment sequence, period, and treatment group was re-evaluated, including any covariate where *P* < 0.05. Data were tested for normality with the Shapiro-Wilk statistic. The effect of treatment was assessed at *P* < 0.05.

The sample size for this study is based on a power calculation for systolic blood pressure. A sample size of 16 participants was determined to detect approximately a 5 mmHg difference in systolic blood pressure (a primary outcome measure) with 80% power and at the 5% significance level. A total number of 20 participants were recruited to allow for dropout.

## Results

**Fig. 1:**
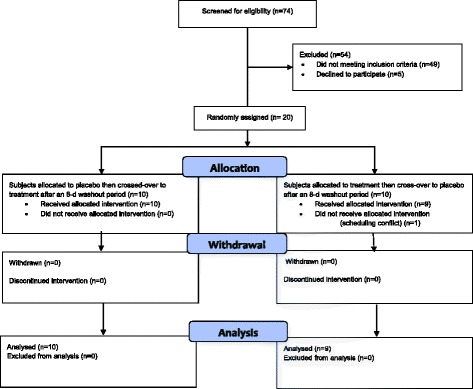
CONSORT diagram for study trial. CONSORT, Consolidated Standards of Reporting Trials

Of the 79 persons who attended the study information meetings, 74 signed an informed consent form, and 74 completed the screening process (60 women, 14 men). Fifty-four individuals were excluded; 49 did not meet the inclusion criteria and 5 declined to participate. All men screened were excluded due to meeting several study exclusion criteria. Ultimately, 20 participants, all women, ages 39 to 64 years of age, were randomly assigned to the treatments. One subject dropped out of the study prior to study initiation (no data was obtained); 19 participants completed all treatment periods (Fig. 1). All participants had at least two risk factors for type 2 diabetes based on the Canadian Diabetes Association [18]. All participants had a BMI >25 kg/m^2^; 84% (*n* = 16) had a waist circumference > 88 cm; 79% (*n* = 15) had a family history of type 1 or type 2 diabetes; 21% (*n* = 4) had a history of gestational diabetes; 47% (*n* = 9) had pre and stage 1 hypertension (blood pressure ≥ 120/80 mmHg and <160/90 mmHg); and 5% (*n* = 1) had hyperlipidemia.

Five percent (*n* = 1) of participants were classified as having prediabetes on the basis of the classification of prediabetes (hemoglobin A1C ≥6.0) from the Canadian Diabetes Association Clinical Practice Guidelines [18]. Seven participants were identified as having insulin resistance (HOMA-IR >2.6), and 12 participants were identified as being insulin sensitive (HOMA-IR ≤2.6) [30]. BMI of the participants (mean, 31.4 kg/m^2^; range, 27 to 37 kg/m^2^) indicated that they were classified as overweight (*n* = 6) or obese (*n* = 13). The baseline physical characteristics of the 19 participants included in the final data are presented in Table 2.

**Table 2:** Characteristics of study participants at baseline^a^

	Total^b^	Range
Age (y)	53 ± 6.3	39–64
Height (cm)	161.5 ± 4.8	154–172
Weight (kg)	81.9 ± 9.4	63–101
Body mass index (kg/m^2^)	31.4 ± 2.9	27–37
Body fat (%)	41.8 ± 3.7	36–49
Waist circumference (cm)	97.9 ± 9.9	86–122
Systolic blood pressure (mm Hg)	126.6 ± 14.0	96–140
Diastolic blood pressure (mm Hg)	81.7 ± 8.7	61–95
Insulin (μU/ml)	9.5 ± 5.8	2.0–22.3
Glucose (mmol/L)	5.4 ± 0.3	4.8–5.9
HOMA-IR	2.3 ± 1.4	0.4–5.7
Hemoglobin A1C	5.5 ± 0.4	4.6–6.1
Cholesterol (mmol/L)	5.1 ± 0.9	3.7–6.5
LDL-Cholesterol (mmol/L)	3.2 ± 0.8	2.0–4.7
HDL-Cholesterol (mmol/L)	1.4 ± 0.3	1.0–2.2
Triacyglycerol (mmol/L)	0.9 ± 0.2	0.5–1.3
Ethnicity (%)		
White, not hispanic	100	

^a^All values are means ± SD

^b^ *n* = 19 females

Mean daily energy intake was 7.99 ± 0.25 kJ (1910 ± 59 kcal) and 8.02 ± 0.25 kJ (1916 ± 59 kcal) for the wild blueberry juice and placebo groups, respectively, and did not differ significantly among the two treatment groups. There were no significant differences in treatment groups on changes in carbohydrate, protein, fat, cholesterol or fiber intake, as determined by the ASA24-Canada-2014 24-h recall method, during the course of the intervention for women participants. In addition, review of 24-h dietary recalls showed an avoidance of high anthocyanin foods throughout the study period. Anthocyanins and other nutrients in the treatment beverages were found to be stable at the end of the study when compared to analyses at the beginning of the study (data not shown). Body weight and anthropometrics did not change significantly throughout the study pre- and post-treatment means 81.9 ± 9.4 kg compared to 81.9 ± 9.4 kg (*P* = 0.856).

Participants were asked to self-monitor compliance to the consumption of the treatment beverages (placebo and wild blueberry juice) on a daily questionnaire. In addition, participants verbally reported consuming the amount of treatment beverages to researchers. Researchers also counted the number of returned beverage containers weekly. The daily questionnaire, subject self-report, and number of beverage containers returned showed an average daily consumption of 240 mL of either the wild blueberry juice or placebo beverage during the treatment periods, which was the prescribed amount.

Systolic and diastolic blood pressures were not significantly different after consumption of the wild blueberry juice for one week compared to the placebo beverage. Systolic blood pressure, however, was lowered by 4.8 mmHg (4%) with consumption of the wild blueberry juice compared to consumption of the placebo beverage (*P* = 0.088). Furthermore, for systolic blood pressure there was a trend for a treatment x status interaction. Participants with pre and stage 1 hypertension experienced a greater lowering by 7.5 mmHg (6%) of systolic blood pressure after wild blueberry juice consumption (*P* = 0.074). Endothelial function of the participants measured by ENDO-PAT2000, expressed as RHI, did not improve significantly (Table 3).

**Table 3:** Effect of 100% wild blueberry juice compared with placebo beverage on blood pressure and endothelial function^a^

Variable	Wild blueberry juice	Placebo beverage	*P*
Systolic blood pressure (mm Hg)^b^	116.0 ± 2.2	120.8 ± 2.2	0.088
Diastolic blood pressure (mm Hg)^b^	79.8 ± 1.4	81.6 ± 1.4	0.243
Systolic blood pressure (mm Hg), pre and stage 1 HTN^c^	120.5 ± 5.8	128.0 ± 5.1	0.074
Diastolic blood pressure (mm Hg), pre and stage 1 HTN^c^	84.1 ± 3.7	86.7 ± 2.9	0.336
Reactive hyperemia index (RHI)^b^	2.23 ± 0.2	2.13 ± 0.2	0.562

^a^All values are lsmeans ± SE. Analysis of covariance appropriate for a two period, two treatment crossover study with baseline values prior to each period; ^b^ *n* = 19, females; ^c^ *n* = 9, females with pre and stage 1 hypertension; RHI, endothelial function

There were no significant differences observed among consumption of the wild blueberry juice and placebo beverage on glucose regulation, surrogate markers of insulin sensitivity, and triglyceride concentrations. In addition, there was no effect on these biomarkers related to insulin resistance status (HOMA-IR > 2.6) of the participants. Fasting plasma glucose concentrations were 5.3 ± 0.1 mmol/L after consumption of wild blueberry juice compared with 5.4 ± 0.1 mmol/L after drinking placebo beverage (*P* = 0.980). Fasting insulin concentrations were not significantly different (9.2 ± 0.8 μU/ml wild blueberry juice compared to 9.6 ± 0.8 μU/ml placebo beverage; *P* = 0.511); HOMA-IR (2.3 ± 0.2 wild blueberry juice compared to 2.3 ± 0.2 placebo beverage; *P* = 0.768) and QUICKI (44.0 ± 1.7 wild blueberry juice compared to 43.0 ± 1.7 placebo beverage; *P* = 0.459) were not significantly different. Triglyceride concentrations were not significantly different (0.9 ± 0.1 mmol/L wild blueberry juice compared with 1.0 ± 0.1 mmol/L placebo beverage; *P* = 0.491). No significant differences were observed between the two treatment beverages in biomarkers of inflammation, adhesion molecules, and oxidative stress. Nitric oxide concentrations were significantly (*P* = 0.039) higher in participants consuming the wild blueberry juice compared with the placebo beverage (Table 4). There was no treatment x status interaction for participants with pre and stage 1 hypertension (2.6 ± 1.4 μM wild blueberry juice compared with 2.3 ± 1.4 μM placebo beverage; *P* = 0.647).

**Table 4:** Effect of 100% wild blueberry juice compared with placebo beverage on biomarkers of inflammation, adhesion molecules, oxidative stress and nitric oxide^a^

Variable	Wild blueberry juice	Placebo beverage	*P*
IL-6 (pg/ml)	0.8 ± 0.07	0.9 ± 0.07	0.203
IL-10 (pg/ml)	0.28 ± 0.02	0.28 ± 0.02	0.659
CRP (ng/ml)	2130.4 ± 52.8	2141.2 ± 53.0	0.887
TNF-α (pg/ml)	2.0 ± 0.4	2.1 ± 0.4	0.399
SAA (ng/ml)	10.1 ± 1.1	10.1 ± 1.1	0.771
sICAM (ng/ml)	305.8 ± 9.6	311.5 ± 9.6	0.551
sVCAM (ng/ml)	969.7 ± 255.5	910.3 ± 256.0	0.665
LDL-oxidation(U/L)	44.5 ± 1.7	44.6 ± 1.7	0.259
Total 8-isoprostanes (pg/ml)	80.1 ± 8.0	80.8 ± 8.0	0.935
Nitric oxide (μM)	4.1 ± 0.4	2.9 ± 0.4	0.039

^a^ *n* = 19, females; all values are lsmeans ± SE. Analysis of covariance appropriate for a two period, two treatment crossover study with baseline values prior to each period; interleukin-6 (IL-6), interleukin-10 (IL-10), high sensitivity C-reactive protein (CRP), tumor necrosis factoralpha (TNF-α), serum amyloid A (SAA), soluble intercellular adhesion molecule-1 (sICAM), and soluble vascular adhesion molecule-1 (sVCAM)

## Discussion

This randomized, single-blind, placebo-controlled, crossover design human intervention study is among the first to assess short-term effects of consuming 240 mL of 100% wild blueberry juice daily on cardiometabolic biomarkers in women having ≥2 risk factors for type 2 diabetes. Results suggest that blood pressure, endothelial function, insulin sensitivity, fasting plasma glucose, serum lipids, inflammation, adhesion molecules, and oxidative stress did not change significantly with wild blueberry juice consumption. However, systolic blood pressure was lowered by 4.8 mmHg (4%) with consumption of the wild blueberry juice compared to participants consuming placebo beverage. In addition, participants with pre and stage 1 hypertension experienced a greater lowering of systolic blood pressure by 7.5 mmHg (6%), after wild blueberry juice consumption. Although these differences were not statistically significant (*P* = 0.088 and *P* = 0.074 respectively; Table 3), the absolute reduction of 4 to 8 mmHg in systolic blood pressure is clinically significant. A reduction of only 3 mmHg may reduce mortality from coronary heart disease and stroke by 5 to 8% [31], while a reduction of 5 mmHg may reduce the risk of a cardiovascular event by approximately 20% over 5 years [32]. Thus, consumption of 100% wild blueberry juice may have a beneficial impact on cardiovascular disease prevalence and mortality.

To our knowledge, three other human clinical trials have shown that daily blueberry consumption lowers blood pressure, in agreement with our findings [6, 7, 33]. These studies all utilized a parallel design and fed freeze-dried blueberry powder as a treatment, not juice, and the duration of the intervention was much longer (6 or 8 weeks). Daily consumption for 8 weeks of 22 g of freeze-dried blueberry powder (equating to approximately 1 cup of fresh whole blueberries providing 103 mg anthocyanins and 186 mg total polyphenols), mixed with 240 mL water, significantly lowered both systolic and diastolic blood pressure by 5.1% and 6.3%, respectively, and increased nitric oxide concentration in postmenopausal women with pre and stage 1 hypertension [7]. This amount of anthocyanins and phenolics is less than our study, but the treatment duration was 8 times longer and the effects on blood pressure are similar. Similarly, Basu et al. evaluated daily consumption for 8 weeks of 50 g of freeze-dried blueberry powder providing 742 mg anthocyanins and 1624 mg total polyphenols, mixed with 480 mL water. This study showed a 6% and 4% lowering of systolic and diastolic blood pressure, respectively, in obese middle-aged men and women with metabolic syndrome [6]. Thus, the effects on systolic blood pressure are similar to our results. A third parallel design study reported that men and women with prehypertensive systolic and/or diastolic blood pressure experienced significantly lowered diastolic blood pressure after consuming 38 g of freeze-dried blueberry powder with the evening meal for six weeks. Anthocyanins and total polyphenols were not reported [27]. We did not find any effects on diastolic blood pressure, possibly due to the short duration of our intervention. On the contrary, three clinical trials showed no effect of blueberries on blood pressure [8, 9, 34]. Two of these studies were randomized with a parallel design and evaluated daily consumption of 45 g of freeze-dried blueberry powder (providing ~600 mg anthocyanins and 1500 mg total polyphenols) mixed as a smoothie with milk and yogurt for 6 weeks. This regimen did not decrease either systolic or diastolic blood pressure in men and women with metabolic syndrome [9]. Riso et al. conducted a randomized, cross-over design clinical trial showing that daily consumption for 6 weeks of 25 g of freeze-dried blueberry powder (providing 375 mg anthocyanins, total polyphenols, not determined) mixed with 250 mL water did not decrease either systolic or diastolic blood pressure, or change nitric oxide levels in men with at least one risk factor for cardiovascular disease [34]. Inconsistent results of these studies are likely due to differences in study design, namely: the amount of freeze-dried blueberries; dose of anthocyanins and total polyphenols; consumption method, either mixed with milk products or water or other interactions within the food matrix; timing of consumption, either once daily or in the morning and evening; lack of control for diet and other nutrients such as fiber; blood pressure measurement and collection protocols; duration of clinical trial and presence or absence of risk factors from the baseline characteristics of the studied populations. Most participants in studies evaluating blood pressure had normal baseline blood pressure levels [8, 9, 33, 34]. Individuals with normal values may be unlikely to have reductions. The lack of power calculation and appropriate sample size for the primary outcome variable, and diastolic and/or systolic blood pressure may also add to the inconsistency. Only two out of the six clinical trials provided an adequate power calculation for blood pressure [7, 8].

In the present study, blood concentrations of nitric oxide increased in our participants (all women) after consumption of wild blueberry juice, providing a potential mechanism for the blood pressure lowering effect. Nitric oxide initiates and preserves vasodilation through a series of biological actions that conclude in the relaxation of smooth muscle cells that line blood vessels. Nitric oxide is released from endothelial cells in the walls of blood vessels, inducing vasodilation, which lowers blood pressure [35]. However, there was no treatment x status interaction for nitric oxide in participants with pre and stage 1 hypertension. Previously, consumption of freeze-dried blueberry powder for 8 weeks was shown to increase nitric oxide concentration in postmenopausal women with pre and stage 1 hypertension [7] but not in men fed for 6 weeks with at least one risk factor for cardiovascular disease [34]. So far, the effects of blueberry feeding on nitric oxide has only been demonstrated in women.

The effect of blueberry juice consumption on endothelial function has not been previously investigated, although some human clinical trials have evaluated the effects of consuming other forms of blueberry on endothelial function with inconsistent results. In an acute study, there was a significant improvement in endothelial function, evaluated by flow-mediated dilation of the brachial artery, at 1–2 h and 6 h after healthy young men consumed freeze-dried wild blueberry powder mixed with 500 mL water (containing 310, 517, and 724 mg anthocyanins, respectively; 766–1791 mg total polyphenols) [14]. These anthocyanin doses are higher than the daily dose in the present study. A longer-term randomized, parallel design clinical trial reported that daily consumption of 45 g of freeze-dried blueberry powder (providing 580 mg anthocyanins and 1547 mg total polyphenols) mixed with dairy products for 6 weeks also improved endothelial function, expressed as RHI, in men and women with metabolic syndrome [8]. Research suggests that the vascular-improving properties of blueberries may be due to the effects of polyphenol metabolites inhibiting NADPH oxidase, which improves nitric oxide bioavailability leading to increased endothelial-dependent vasodilation [14, 36]. However, not all studies have demonstrated an improvement in endothelial function or enhancement of nitric oxide [34].

There is encouraging preclinical evidence that blueberries may improve glucose management, even though our study produced negative results. Blueberry extracts reduced plasma glucose 6 h after ingestion in mice [37]. Blueberry polyphenols stabilized in soybean flour also reduced glucose intolerance in mice and decreased glucose production in rat hepatocytes [38]. Further, wild blueberry extracts were found to induce beta-cell proliferation in vitro [39]. In the present study, there were no significant changes in fasting glucose, insulin, and triglyceride concentrations after consumption of the wild blueberry juice; there was also no effect on surrogate markers of insulin resistance, HOMA-IR, and insulin sensitivity, QUICKI. In agreement with our study, a recent meta-analysis showed that 100% fruit juice may have no overall effect on fasting glucose and insulin concentrations in participants with obesity, metabolic syndrome, hypertension, and type 2 diabetes [40]. Also, previous human clinical trials have shown that freeze-dried blueberry powder had no significant effect on glucose metabolism, surrogate markers of insulin sensitivity and triglycerides, in adults with metabolic syndrome and risk factors for cardiovascular disease [6, 8, 34]. However, daily consumption of freeze-dried blueberry powder for 6 weeks improved insulin resistance as determined by hyperinsulinemic-euglycemic clamp in men and women with insulin resistance [9]. More research is required to differentiate between the effects of the whole blueberry fruit versus 100% fruit juice on human health and risk factors for type 2 diabetes.

Circulating markers of inflammation, e.g., IL-6, a proinflammatory cytokine, s-ICAM, an adhesion molecule, and CRP, a liver acute phase reactant, have been identified as potential predictors of present and future risk of chronic disease in individuals [41, 42]. We found that wild blueberry juice had no effect on any biomarkers of inflammation in women at risk for type 2 diabetes. Human clinical trials have shown no significant effects of freeze-dried blueberry powder on CRP, IL-6, TNF-α, and sVCAM in patients with metabolic syndrome, hypertension, and other risk factors for cardiovascular disease [6, 9, 34], and the effects of blueberry juice have not been examined previously. Further research may be warranted, such as dose-response studies to determine inflammation endpoints in those at risk for chronic disease.

Risk factors for type 2 diabetes, such as obesity and insulin resistance, may be significant determinants of oxidative stress [43]. Isoprostanes, produced mainly through the non-enzymatic oxidation of arachidonic acid by reactive oxygen species, are recognized biomarkers of in vivo lipid peroxidation, and the production of isoprostanes is increased in the presence of oxidative stress [44]. An additional measure of oxidative stress is LDL-oxidation for also assessing lipid oxidation [45]. In the present study, we observed no effects on total 8-isoprostanes and LDL-oxidation concentrations with consumption of the wild blueberry juice. Few human clinical trials have evaluated the effects of blueberries on oxidative stress in adults at risk for disease. Basu et al. showed that after 8 weeks, consumption of freeze-dried blueberry powder mixed with water, lowered LDL-oxidation concentrations in obese men and women with metabolic syndrome [6]. Similarly, Riso et al. reported that consumption of freeze-dried blueberry powder mixed with water for 6 weeks lowered endogenously oxidized DNA bases and H_2_O_2_-induced DNA damage without affecting erythrocyte superoxide dismutase activity in men with risk factors for cardiovascular disease [34]. Our findings may be due to the short study duration.

The strengths and limitations of our study design warrant consideration. First, to our knowledge this is the first study that evaluated the effects of 100% wild blueberry juice compared to a placebo on cardiometabolic biomarkers with a randomized, crossover, single blind design; this is one of the most powerful designs for evaluating the efficacy of dietary treatments [46]. Second, several measures of adherence to the study treatments were made. In addition, dietary intake was evaluated and appeared to stay constant throughout the 3-week study, along with subject body weight. There were, however, several limitations to the study. Although this was an exploratory study, the small sample size was limiting and the study may not have been adequately powered to detect existing differences in some biomarkers. Though our study intervention of 7 days was relatively short, several previous studies have shown that short term interventions with berries may produce significant changes in glucose metabolism and other biomarkers of disease [14–17, 47–49]. However, the short duration of our study may not be sufficient to produce biological effects in several of the outcome variables investigated in this study; longer clinical trials are necessary. Also, results of the study are only valid for women at risk for type 2 diabetes.

## Conclusions

The results of the present study indicate that short-term consumption of 240 mL daily of wild blueberry juice may promote cardioprotective effects, by improving systolic blood pressure, possibly through nitric oxide production, in adults at risk for type 2 diabetes. This outcome warrants longer-term human studies of blueberries, including defined amounts of either the whole fruit or juice, to clarify whether polyphenol-rich foods can be efficacious for improving cardiometabolic biomarkers in individuals at risk for type 2 diabetes.

## Abbreviations

ANCOVA: Analysis of covariance
ASA24: Automated Self-Administered 24-h Recall
BMI: Body mass index
CRP: High-sensitivity C- reactive protein
DNA: Deoxyribonucleic acid
H_2_O_2_: Hydrogen peroxide
HDL: High density lipoprotein
HOMA-IR: Homeostasis model assessment of insulin resistance
IL-10: Interleukin-10
IL-6: Interleukin-6
LDL: Low density lipoprotein
NADPH: Nicotinamide adenine dinucleotide phosphate
PAT: Peripheral artery tone
QUICKI: Quantitative insulin sensitivity check index
RHI: Reactive hyperemia index
SAA: Serum amyloid A
sICAM: Soluble intercellular adhesion molecule-1
sVCAM: Soluble vascular adhesion molecule-1
TNF-α: Tumor necrosis factor-alpha
